# Genetic characterization of cassava (*Manihot esculenta* Crantz) genotypes using agro-morphological and single nucleotide polymorphism markers

**DOI:** 10.1007/s12298-019-00740-x

**Published:** 2019-12-23

**Authors:** Kumba Y. Karim, Beatrice Ifie, Daniel Dzidzienyo, Eric Y. Danquah, Essie T. Blay, Jim B. A. Whyte, Peter Kulakow, Ismail Rabbi, Elizabeth Parkes, Lucky Omoigui, Prince E. Norman, Peter Iluebbey

**Affiliations:** 1 Sierra Leone Agricultural Research Institute, Tower Hill, Freetown, PMB 1313 Sierra Leone; 2 International Institute of Tropical Agriculture, Ibadan, PMB 5320 Nigeria; 3 West Africa Centre for Crop Improvement, College of Basic and Applied Sciences, University of Ghana, P.O. Box LG 30, Legon, Accra, Greater Accra Ghana

**Keywords:** Cassava, Genetic diversity, Morphological traits, SNP markers

## Abstract

Dearth of information on extent of genetic variability in cassava limits the genetic improvement of cassava genotypes in Sierra Leone. The aim of this study was to assess the genetic diversity and relationships within 102 cassava genotypes using agro-morphological and single nucleotide polymorphism markers. Morphological classification based on qualitative traits categorized the germplasm into five different groups, whereas the quantitative trait set had four groups. The SNP markers classified the germplasm into three main cluster groups. A total of seven principal components (PCs) in the qualitative and four PCs in the quantitative trait sets accounted for 79.03% and 72.30% of the total genetic variation, respectively. Significant and positive correlations were observed between average yield per plant and harvest index (r = 0.76***), number of storage roots per plant and harvest index (r = 0.33*), height at first branching and harvest index (0.26*), number of storage roots per plant and average yield per plant (r = 0.58*), height at first branching and average yield per plant (r = 0.24*), length of leaf lobe and petiole length (r = 0.38*), number of leaf lobe and petiole length (r = 0.31*), width of leaf lobe and length of leaf lobe (r = 0.36*), number of leaf lobe and length of leaf lobe (r = 0.43*), starch content and dry matter content (r = 0.99***), number of leaf lobe and root dry matter (r = 0.30*), number of leaf lobe and starch content (r = 0.28*), and height at first branching and plant height (r = 0.45**). Findings are useful for conservation, management, short term recommendation for release and genetic improvement of the crop.

## Introduction

Cassava (*Manihot esculenta* Crantz) is a very important root crop, containing high carbohydrate levels, used for human consumption, animal feed and industrial applications (Sánchez et al. 2009). The starchy storage roots of cassava have become the most important source of dietary energy in sub-Saharan Africa (SSA) as they provide more returns per unit of input than any other staple crop (Fregene et al. 2000; Scott et al. 2000; Nassar 2005). Cassava is a hardy plant that survives in poor soils with low fertility, relatively producing higher yields than other root and tuber crops (Temegne et al. 2015). However, cassava genotypes respond differently to diverse environmental (soil, climate) and biotic factors (Dixon et al. 2002).

In Sierra Leone, dearth of information on the extent of genetic variation within the breeding population of cassava limits the development of superior cassava genotypes. Determination of the genetic variation in breeding population facilitates identification of useful genetic divergence imperative for cassava population improvement. Genetic divergence in breeding population is evaluated by genetic markers (Andrade et al. 2017). Genetic markers such as agro-morphological markers had been used frequently in preliminary studies because they are fast and easy approach for assessing the extent of diversity among germplasm (Asare et al. 2011). Some of these morphological traits revealed the true diversity as perceived by farmers (Mckey et al. 2001; Pinton and Emperaire 2001). Elias et al. (2001) also reported that morphological traits have a heritable genetic variation. As knowledge in scientific research progressed, molecular markers were noted to unravel the genetic constitution and significance of traits through DNA fingerprinting, gene link detection, identification of genotypes, gene introgression, germplasm characterization, phylogenetic analysis, and indirect selection of agronomic traits (Souza 2015; Andrade et al. 2017). Such knowledge underpins the use of appropriate and reliable agro-morphological descriptor and molecular markers for the evaluation of genetic diversity (Fukuda and Guevara 1998).

Quantitative and qualitative morphological traits have been used for systematic identification of genotypes, species and genera of some crops (Smykal et al. 2008). Qualitative traits are usually controlled by few genes with major effects. These traits are easily observable, thereby making differentiation and identification of genotype easier. Conversely, quantitative traits are controlled by many minor genes with complex inheritance. These traits are more affected by environmental effects and developmental stage of the crop.

Genetic diversity studies using morphological traits alone are sometimes limited by the environment and genotype by environment interaction effects (Collard et al. 2005). These limitations may not permit the accurate detection of duplicates by morphological classification technique alone. Collard et al. (2005) reported that the use of molecular markers may permit the detection of genetic differences among closely related genotypes. Characterization of accessions may, therefore, be more reliable if molecular markers are closely associated with morphological traits. Various DNA markers have been utilized to assess genetic diversity in cassava germplasm (Okogbenin et al. 2012). These include restriction fragment length polymorphism (RFLPs) (Beeching et al. 1993), random amplified polymorphic DNA (RAPDs) (da Silva et al. 2015), amplified fragment length polymorphism (AFLP) (Fregene et al. 2000), simple sequence repeats (SSRs) (Asare et al. 2011) and single nucleotide polymorphism (SNP) markers (Kawuki et al. 2009). Of the above marker systems, SSR and SNP markers are among competitive markers for diversity studies. However, microsatellites may be limited by the presence of stutter bands that produce quasi-scoring in ladders lacking prominent bands thereby making scoring difficult (Park et al. 2009) and poor transferability across species (Grattapaglia and Kirst 2008). Single nucleotide polymorphisms are more easily assayed per locus compared to microsatellites. The SNPs are the most abundant marker system in plant, animal, and microorganism genomes and are considered as the new generation molecular marker for various applications. The SNPs are useful in detecting and distinguishing specific genetic variations even in a low diversity species (Ferri et al. 2010). The use of SNPs has accelerated the pace of genetic diversity research and gains in selection rather than using the conventional technique alone. Thus, the objective of the present study was to assess the genetic diversity and relationships within cassava germplasm using agro-morphological and single nucleotide polymorphism markers.

## Materials and methods

### Plant material, experimental design and plot layout

The trials were established in-field at the Njala Agricultural Research Centre (NARC) experimental site, southern Sierra Leone in the 2015/2016 cropping season. Njala is situated at an elevation of 50 m above sea level, 8° 06′ N latitude and 12° 06′ W longitude. A total of 102 cassava genotypes comprising 82 white and 20 yellow accessions were evaluated to determine the extent of genetic diversity within the breeding population (Table 1). The experiment was laid out in a 6 × 17 alpha lattice design with three replications. Stem cuttings measuring 30 cm in length each were planted on the crest of ridges at 1 × 1 m spatial arrangement. No fertilizer or herbicide was applied. Hand weeding was done when necessary.

**Table 1:** Storage root flesh attributes of 102 cassava genotypes used for the genetic characterization study

Genotype	Storage root flesh color	Genotype	Storage root flesh color	Genotype	Storage root flesh color
TR0991	White	TR0188	White	TR1523	White
TR1827	White	TR1782	White	TR0218	White
TR1036	White	TR1069	White	TR0043	White
TR1013	White	TR0075	White	SLICASS6	White
TR0038	White	TR0575	White	TR0142	White
TR0768	White	TR1696	White	TR0813	White
TR0519	White	TR0127	White	TR0971	White
TR0523	White	TR0329	White	TR0593	White
TR0092	White	TR0864	White	TR0589	White
TR1198	Yellow	TR0302	White	TR0345	White
TR1028	Yellow	TR1005	White	TR0135	White
TR0949	Yellow	TR1162	White	TR1436	White
TR0300	Yellow	TR0159	White	TR1326	Yellow
TR0356	White	TR0006	White	TR0454	White
TR1769	White	TR0255	White	TR1736	White
TR0024	White	TR0435	White	TR0724	White
TR0275	White	TR0428	White	TR0310	White
TR0694	Yellow	TR1791	White	TR0417	White
TR0912	White	TR0288	White	TR0120	White
TR0105	White	TR1716	Yellow	TR0164	White
TR0171	White	TR0432	White	TR0263	White
TR0488	White	TR0297	Yellow	TR0590	White
SLICASS4	White	TR1167	White	TR0382	Yellow
TR0746	White	TR1175	White	TR0453	White
TR0740	White	TR0821	White	TR1154	White
TR0204	White	TR0657	White	TR1097	White
TR1518	Yellow	TR1035	Yellow	TR0545	White
TR0064	White	TR1618	Yellow	TR1041	Yellow
TR0455	White	TR0613	White	TR0745	White
TR0779	White	TR0358	White	TR0017	White
TR0820	White	TR1796	Yellow	TR0591	White
TR0915	White	TR1288	White	TR1159	White
TR1824	White	TR0667	White	TR1776	White
TR0812	White	TR0256	White	COCO	White

### Agro-morphological data collection

A total of 22 agro-morphological traits comprising 11 quantitative and 11 qualitative traits were evaluated (Table 2) based on the agro-morphological descriptor of cassava described by Fukuda et al. (2010).

**Table 2:** Qualitative and quantitative traits used to characterize 102 cassava genotypes

SN	Trait descriptor	Trait acronym	Score code	Sampling time
Qualitative traits				
1	Color of leaf vein	CLV	3 = green; 5 = reddish green in < half of lobe; 7 = reddish green in > half of lobe; 9 = all red	6 MAP
2	Root taste	RT	1 = sweet; 2 = intermediate; 3 = bitter	12 MAP
3	Cassava mosaic disease	CMD	1 = no visible symptom of disease; 2 = mild; 3 = low; 4 = intermediate; 5 = high	6 MAP
4	Color of root pulp	CRP	1 = white; 2 = cream; 3 = yellow; 4 = orange; 5 = pink	12 MAP
5	Lobe margins	LM	3 = smooth; 7 = winding	6 MAP
6	Ease of peeling	EP	1 = easy; 2 = difficult	12 MAP
7	Leaf color	LC	3 = light green; 5 = dark green; 7 = purple green; 9 = purple	6 MAP
8	Color of apical leaves	CAL	3 = light green; 5 = dark green; 7 = purplish green; 9 = purple	3 MAP
9	Root shape	RS	1 = conical; 2 = conical-cylindrical; 3 = cylindrical; 4 = irregular	12 MAP
10	Shape of central leaflet	SCL	1 = ovoid; 2 = elliptical-lanceolate; 3 = obovate-lanceolate; 4 = oblong-lanceolate; 5 = lanceolate; 6 = straight; 7 = pandurate; linear-piramidal; linear-pandurate; linear-hostatilobalate	6 MAP
11	External color of storage root	ECSR	1 = white or cream; 2 = yellow; 3 = light brown; 4 = dark brown	12 MAP
Quantitative traits				
12	Number of storage roots/plant	NSR	Count	12 MAP
13	Harvest index	HI	Derived estimate	12 MAP
14	Root yield per plant	RYPP	Derived estimate	12 MAP
15	Root dry matter content (%)	RDMC	Direct measurement	12 MAP
16	Starch content (%)	STC	Direct measurement	12 MAP
17	Number of leaf lobe	NLL	Count	6 MAP
18	Petiole length	PL	Direct measurement using meter rule	6 MAP
19	Plant height	PH	Direct measurement using meter rule	12 MAP
20	Length of leaf lobe	LLL	Direct measurement using meter rule	6 MAP
21	Width of leaf lobe	WLL	Direct measurement using caliper	6 MAP
22	Height at first branching	HFB	Direct measurement using meter rule	12 MAP

Harvest index (HI) was calculated at harvest as the ratio of fresh root yield to the total fresh biomass (weight of roots and weight of above ground biomass).

Starch extraction was done at harvest using a method described by Benesi (2005).

Starch content was calculated as:

$$ {\text{Starch content}} (\%) = {\frac{{\text{DSW}}}{{\text{FM}}}} \times 100 $$

where DSW is the weight of dried starch and FM is the weight of fresh tuber.

Root dry matter content (RDMC) determination was done at harvest by selecting three representative roots from the bulk of roots harvested from 5 plants. Cassava roots were washed and shredded into pieces. A standard measure of 100 g weight of the fresh samples was taken and oven dried with forced drought oven. Samples were reweighed again to obtain a constant weight after 72 h at 65–70 °C (Fukuda et al. 2010).

### Molecular characterization

DNA was extracted at the International Institute of Tropical Agricultural Bioscience Laboratory IITA, Ibadan, Nigeria using the method proposed by Dellaporta et al. (1983) with a slight modification described by Rabbi et al. (2014). Freshly harvested apical leaves of about 200 mg of each accession were used. Grinding of the leaf samples was done in a 1.2 ml extraction tube using 400 µl extraction buffer and then placed in a 65 °C water-bath for 25 min with gentle shaking. The tube was removed from the water bath and allowed to cool for 5–10 min. Proteins and polysaccharides were precipitated by adding 200 µl of ice-cold 5 M potassium acetate and then mixed by gentle inversions (this was placed on ice for 20 min). About 350 µl chloroform:isomyl alcohol was added (24:1) to the content and mixed gently with continuous rocking and centrifuged at 4000 *g* for 10 min. This was followed by the addition of RNase. The crude pellets were precipitated by transferring the upper layer to a new tube. One volume (400 µl) of ice-cold isopropanol was added and mixed gently for about 2–3 min and then chilled in − 20 °C freezer for 10 min to enhance DNA precipitation. It was then centrifuged at 4500 *g* for 20 min and the supernatant was carefully discarded.

### SNP genotyping

For SNP genotyping, about 50 µl concentrated DNA sample of each sample was sent to Cornell University for genotyping-by-sequencing analysis. The GBS was determined as described by Elshire et al. (2011) and sequenced at the Institute of Genomic Diversity at Cornell University using the Illumina HiSeq 2500. The raw HapMap file from Cornell University was first converted to a Variant call format (VCF) for the analysis using perl programming language and TASSEL 5.0 (Bradbury et al. 2007; Elshire et al. 2011). The VCF file was filtered for missing value and polymorphic SNPs with quality parameter and a call rate greater than 80%, depth > 95%, and minor allele frequency of 0.01. The SNPs with MAF values less than 0.01 and loci with more than 40% missing SNP marker data were considered non-informative and were removed. Of the 8600 SNPs subjected to filtering, 5600 informative SNP markers were retained for genetic diversity study.

### Data analysis

#### Qualitative and quantitative phenotypic data analysis

The genetic variation among the studied genotypes for agro-morphological traits was explored using multivariate analysis technique. Multivariate analysis of the 102 × 11 qualitative data matrix and 102 × 11 quantitative data matrix comprising of principal component analysis (PCA) were performed separately in SAS 9.4 software version. In the PCA, Eigen-values and load coefficient values were generated from the data set. The relevance of trait contribution to the variation accounted by each principal component was based on the absulute eigenvector arbitrary cutoff value of 0.30 (Richman 1988). The PCA and correlation matrices were used to determine the relationships among the traits. The organization and structure of the morphological variability were visualized using the Ascending Hierarchical Clustering (AHC) to plot a dendrogram.

### Molecular data analysis

The genetic analysis package Power Marker version 3.0 (Liu and Muse 2005) was used to generate pairwise distance-based hierarchical clustering.

## Results

### Frequency distribution of accessions according to qualitative traits

**Fig. 1:**
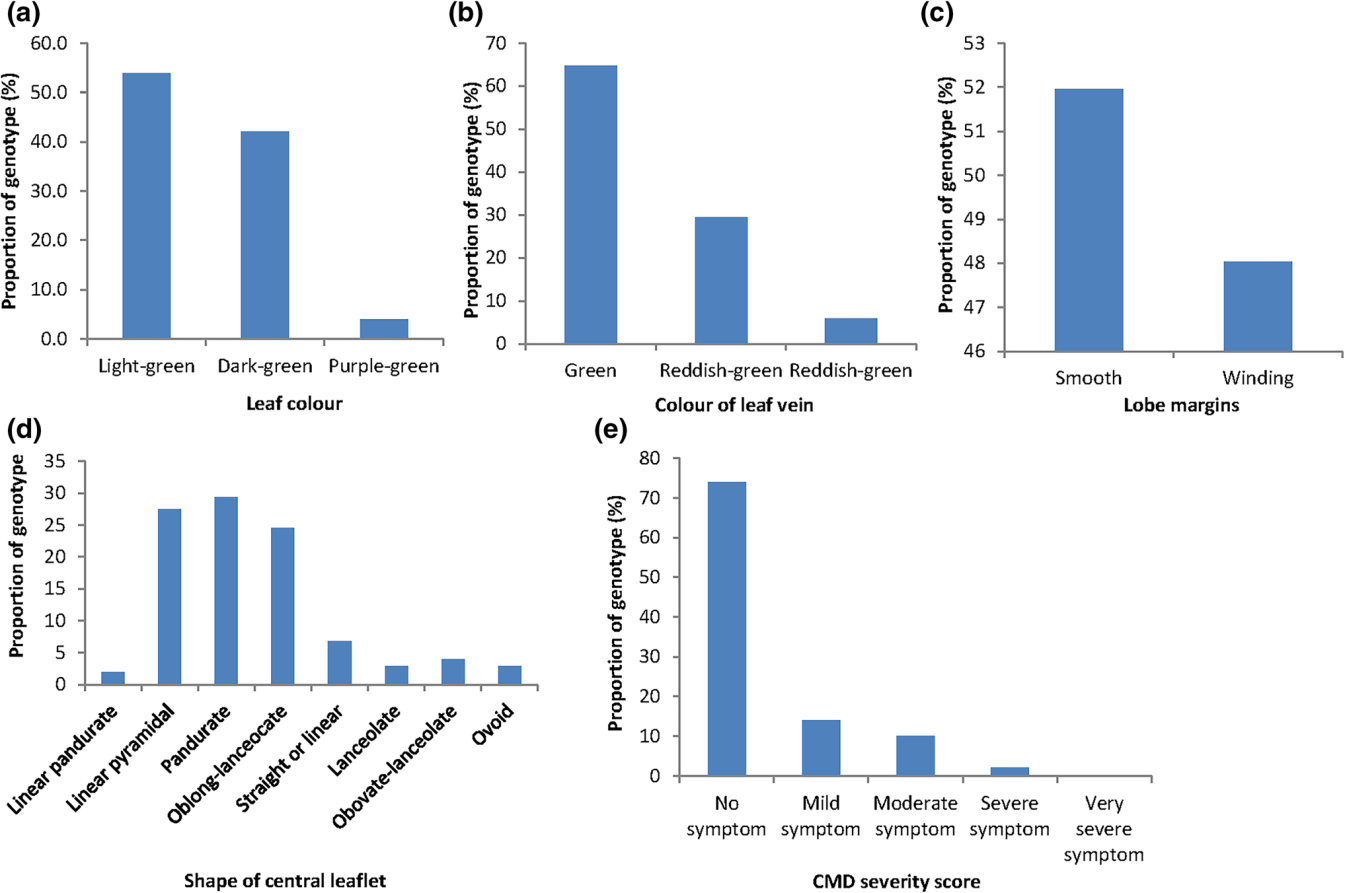
Percent distribution of **a** leaf color; **b** color of leaf vein; **c** lobe margins; **d** shape of central leaflet; and **e** cassava mosaic disease among 102 genotypes of cassava

**Fig. 2:**
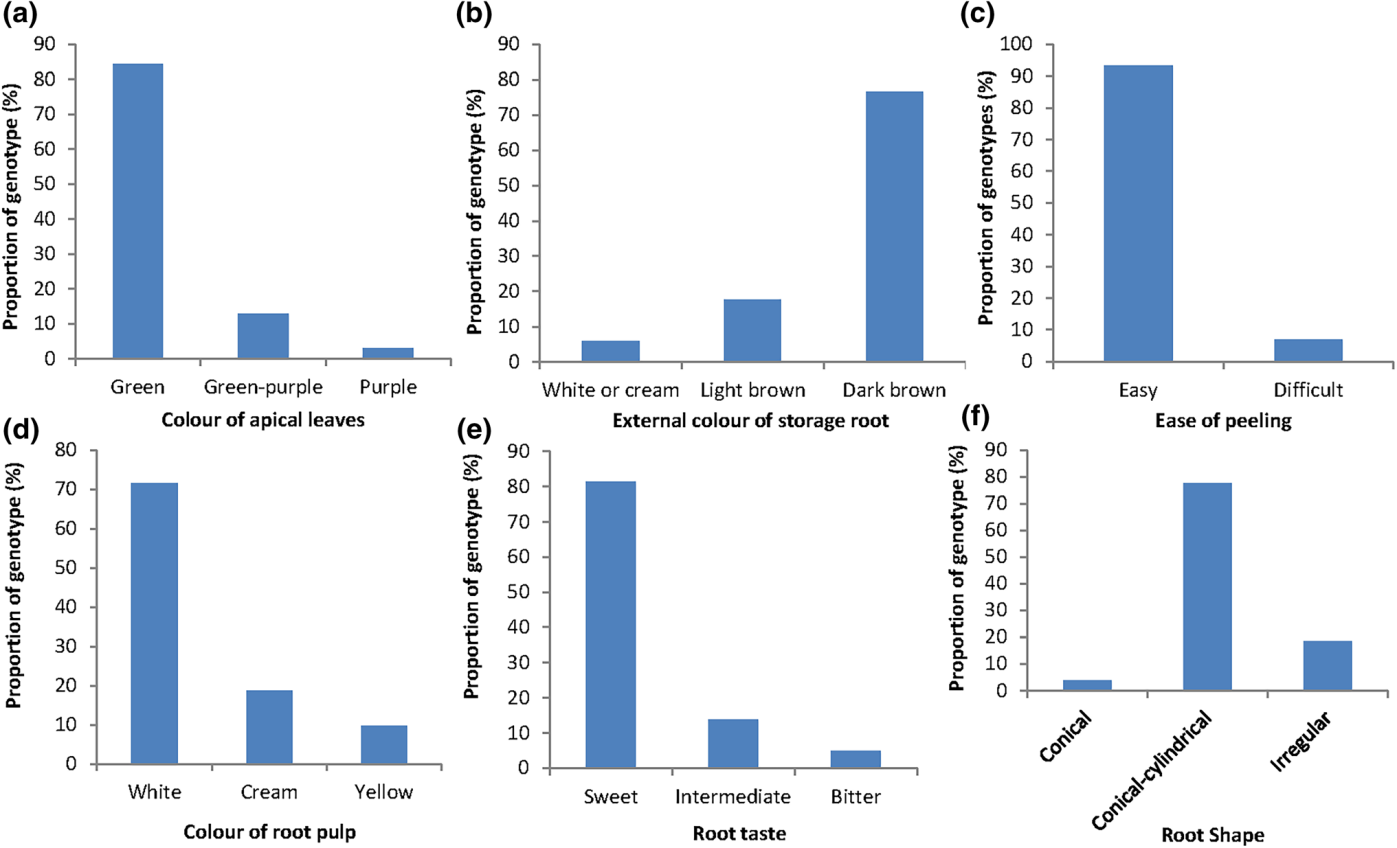
Percent distribution of **a** color of apical leaves; **b** external color of storage root; **c** ease of peeling; **d** color of root pulp; **e** root taste; and **f** root shape among 102 genotypes of cassava

Frequency distributions of the qualitative traits are presented in Figs. 1 and 2. Genetic variability was observed among the 102 cassava accessions for all of the variables evaluated. The results showed that 53.9% of the accessions exhibited light-green leaves, 42.2% had dark-green leaves and 3.9% had purple-green leaves (Fig. 1a). About 67% of the accessions had green leaf vein, 27% had reddish-green in more than half of lobe and 6% had reddish-green in less than half of the lobe (Fig. 1b). Lobe margin of 52% of the accessions was smooth, while 48% had winding lobe margin (Fig. 1c). The shapes of the central leaflet of the accessions were 2.0% linear pandurate, 27.5% linear pyramidal, 29.4% pandurate, 24.5% oblong-lanceolate, 6.9% straight or linear, 2.9% lanceolate, 3.9% obovate-lanceolate and 2.9% ovoid (Fig. 1d). The accessions exhibited 74.0% no symptom, 14.0% mild symptom, 10.0% moderate symptom, 2.0% severe symptom and 0% very severe symptom of cassava mosaic disease severity (Fig. 1e).

In terms of color of apical leaves, 84.3%, 12.7% and 2.9% genotypes had light green, green purple and purple apical leaves, respectively (Fig. 2a). For external color of storage roots, 5.9% of the accessions had white or cream, 17.6% light brown and 76.5% dark brown storage roots (Fig. 2b). Approximately 90.1% of accessions were easy to peel while 9.8% were difficult to peel (Fig. 2c). About 70.6% of the accessions had white root pulp, while 19.6% had cream root pulp and 9.8% had yellow root pulp (Fig. 2d). About 79.4% of the accessions had sweet root taste, 14.7% were classified as intermediate and 5.9% had bitter root taste (Fig. 2e). The accessions comprised of three root shapes including conical (3.9%), conical-cylindrical (77.5%) and irregular (18.6%) (Fig. 2f).

### Principal component analysis of qualitative characters

The eigenvalues and percentage variations of the principal component analysis are presented in Table 3. Seven principal components that accounted for 79.03% of the total variation among the genotypes were identified. The first PC axis with eigenvalue of 1.73 accounted for 15.76% of the total variation where the second, third and the fourth PC axes with eigenvalues of 1.70, 1.35 and 1.14 accounted for 15.43%, 12.24% and 10.38% of the total variation, respectively. The fifth, sixth and seventh PC axes with eigenvalues of 0.99, 0.97 and 0.83 accounted for 8.99%, 8.84% and 7.43% of the total variation, respectively.

**Table 3:** Principal component analysis, eigenvalues and percentage variation of eleven qualitative traits of 102 cassava genotypes

Traits	Prin1	Prin2	Prin3	Prin4	Prin5	Prin6	Prin7
RT	**0.43**	− **0.37**	− 0.04	0.22	**0.32**	0.09	− 0.25
ECSR	− 0.14	0.26	− **0.32**	**0.52**	− 0.05	**0.41**	0.02
CRP	**0.48**	0.10	0.12	− **0.41**	0.06	− 0.02	**0.48**
ES	**0.47**	− 0.22	**0.33**	0.29	0.12	0.15	− 0.18
CLV	− 0.17	0.00	**0.51**	**0.36**	− 0.27	0.02	0.21
CMD	− 0.18	0.13	0.19	**0.33**	**0.67**	− **0.31**	**0.44**
RS	− **0.41**	− 0.12	0.21	− **0.30**	**0.36**	− 0.14	− **0.46**
LM	− 0.21	− 0.18	0.01	− 0.28	**0.30**	**0.78**	0.27
LC	0.22	**0.60**	0.03	− 0.12	0.05	0.01	− 0.07
CAL	0.12	**0.50**	− 0.07	0.03	**0.33**	0.13	− 0.32
SCL	0.03	− 0.23	− **0.65**	0.08	0.15	− 0.20	0.19
Eigenvalue	1.73	1.70	1.35	1.14	0.99	0.97	0.82
Proportion of variance (%)	15.76	15.43	12.24	10.38	8.99	8.84	7.43
Cumulative variance (%)	15.76	31.19	43.43	53.81	62.80	71.64	79.07

^Values in bold represent significant traits in the various principal components, *RT* root taste, *ECSR* external color of storage root, *CRP* color of root pulp, *ES* ease of peeling, *CLV* color of leaf vein, *CMD* cassava mosaic disease, *RS* root shape, *LM* lobe margins, *LC* leaf color, *CAL* color of apical leaves, *SCL* shape of central leaflet^

The first principal component with reference to its high factor loadings was positively associated with traits such as root taste, color of root pulp, ease of peeling, and root shape. The second PC was associated with leaf and storage root characteristics (root taste, leaf color, and color of apical leaves); the third PC was associated with external color of storage root, ease of peeling, color of leaf vein and shape of central leaf lobe, while the fourth PC was associated with traits related to storage root characteristics (color of root pulp, external color of storage root, and root shape) color of leaf vein and cassava mosaic disease. The fifth PC was associated with characteristics such as root taste, cassava mosaic disease, root shape, lobe margin and color of apical leaves, the sixth PC was also associated with external color of storage root, cassava mosaic disease and lobe margin and the seventh PC was also associated with storage (root color of root pulp and root shape) and cassava mosaic disease.

### Genetic relationship among 102 cassava genotypes using 11 qualitative traits

**Fig. 3:**
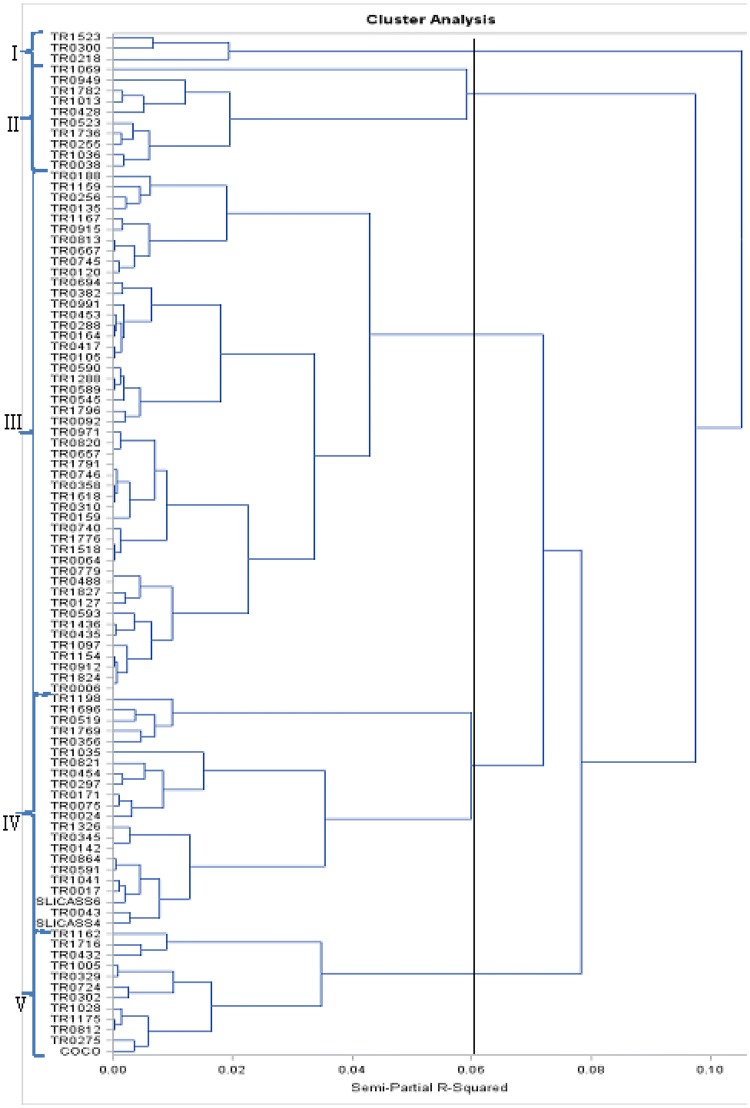
Dendrogram showing relationships among 102 genotypes of cassava classified by Ward method using eleven 11 qualitative agro-morphological traits

The hierarchical classification of qualitative traits grouped genotypes into five classes almost with the same characteristics as a function of the variables (Fig. 3). The genetic similarity for the 11 qualitative traits ranged from zero to one with a mean similarity of 0.10. The cassava genotypes were grouped into five distinct clusters at 0.06 similarities. Groups III, IV and V have a high number of genotypes with 55, 22 and 12, respectively. Ten and 3 individuals were in clusters II and I, respectively (Fig. 3).

### Mean values and correlation coefficients for the eleven quantitative traits

The mean values for harvest index, root yield per plant, root dry matter content, number of storage roots and starch content were 0.5, 1.6 kg, 30.9%, 7.5 and 23.9%, respectively (Table 4). Significant and positive correlations were observed between root yield per plant and harvest index (r = 0.76***), number of storage roots per plant and harvest index (r = 0.33*), height at first branching and harvest index (0.26*), number of storage roots per plant and root yield per plant (r = 0.58*), height at first branching and root yield per plant (r = 0.24*), length of leaf lobe and petiole length (r = 0.38*), width of leaf lobe and petiole length (r = 0.23*), number of leaf lobe and petiole length (r = 0.31*), width of leaf lobe and length of leaf lobe (r = 0.36*), number of leaf lobe and length of leaf lobe (r = 0.43*), starch content and root dry matter content (r = 0.99***), number of leaf lobe and root dry matter content (r = 0.30*), number of leaf lobe and starch content (r = 0.28*), and height at first branching and plant height (r = 0.45**) (Table 5). Conversely, significant and negative associations were noted between height at first branching and length of leaf lobe (− 0.27*), and between height at first branching and width of leaf lobe (− 0.21*).

**Table 4:** Probability values, means and coefficient of variation of quantitative traits of 102 cassava genotypes

Source	HI	AYPP	PL	LLL	WLL	RDMC	NSR	STC	PH	NLL	HFB
Genotype	< .0001	< .0001	< .0001	< .0001	0.9709	0.214	< .0001	0.3192	0.7652	< .0001	0.0098
Mean	0.45	1.56	13.5	10.6	4.4	30.9	7.5	23.9	149.1	5.8	58.9
CV	19.1	32.7	20.9	11.6	100.4	8.8	23.1	11.8	17.55	17.3	55.1

^Significant at alpha = 0.05. *HI* harvest index, *AYPP* average yield per plant (kg), *PL* petiole length (cm), *LLL* length of leaf lobe (cm), *WLL* width of leaf lobe (cm), *RDMC* root dry matter content (%), *NSR* number of storage roots (count), *STC* starch content (%), *PH* plant height (cm), *HFB* height at first branching (cm), *NLL* number of number of leaf lobe^

**Table 5:** Correlation coefficients among 11 quantitative traits of 102 cassava genotypes

	HI	RYPP	PL	LLL	WLL	RDMC	NSR	STC	PH	HFB	NLL
HI	1.00										
RYPP	0.76***	1.00									
PL	0.01	0.11	1.00								
LLL	0.14	0.23	0.38*	1.00							
WLL	− 0.19	− 0.01	0.23*	0.36*	1.00						
RDMC	0.08	0.00	− 0.04	0.01	− 0.16	1.00					
NSR	0.33*	0.58**	0.14	0.19	0.01	0.03	1.00				
STC	0.08	0.00	− 0.05	0.00	− 0.16	0.99***	0.05	1.00			
PH	0.16	0.03	0.06	− 0.18	− 0.17	0.00	− 0.03	− 0.03	1.00		
HFB	0.26*	0.24*	− 0.04	− 0.27*	− 0.21*	0.06	0.08	0.05	0.45**	1.00	
NLL	0.05	− 0.03	0.31*	0.43**	0.19	0.30*	0.11	0.28*	0.07	− 0.03	1.00

^Significant at alpha = 0.05. *HI* harvest index, *RYPP* root yield per plant (kg), *PL* petiole length (cm), *LLL* length of leaf lobe (cm), *WLL* width of leaf lobe (cm), *RDMC* root dry matter content (%), *NSR* number of storage roots (count), *STC* starch content (%), *PH* plant height (cm), *HFB* height at first branching (cm), *NLL* number of leaf lobe^

### Representation of variables of quantitative traits

The result revealed that the four main components accounted for 72.30% of the total variation among the genotypes. The first factorial plane contains 22.18% of the variance. The variables that significantly correlated with axis 1 are: harvest index (47%), root yield per plant (49%), root dry matter content (30%), number of storage roots (40%), and starch percentage (30%). The variables that were significantly correlated with axis 2 are: petiole length (37%), length of leaf lobe (51%), width of leaf lobe (47%) and height at first branching (− 37%). The variables significantly related to axis 3 are: root yield per plant (− 35%), root dry matter content (55%), starch content (55%) and number of leaf lobes (30%). The variables significantly correlated with axis 4 are: petiole length (40%), plant height (64%), height at first branching (40%) and number of leaf lobe (36%) (Table 6).

**Table 6:** Principal component analysis, eigenvalues, percentage variation and cumulative variance of eleven quantitative traits of 102 cassava genotypes

Traits	Prin1	Prin2	Prin3	Prin4
HI	**0.47**	− 0.11	− 0.28	− 0.13
RYPP	**0.49**	0.02	− **0.35**	− 0.23
PL	0.17	**0.37**	0.00	**0.40**
LLL	0.24	**0.51**	0.06	0.02
WLL	− 0.05	**0.47**	0.03	0.10
RDMC	**0.30**	− 0.22	**0.55**	− 0.07
NSR	**0.40**	0.10	− 0.20	− 0.21
STC	**0.30**	− 0.21	**0.55**	− 0.10
PH	0.10	− 0.27	− 0.14	**0.64**
HFB	0.19	− **0.37**	− 0.20	**0.40**
NLL	0.26	0.26	**0.30**	**0.36**
Eigenvalue	2.44	2.14	2.07	1.31
Proportion of variance (%)	22.18	19.42	18.83	11.87
Cumulative variance (%)	22.18	41.60	60.43	72.30

^Figures in bold represent significant traits in the various principal components; *HI* harvest index, *RYPP* root yield per plant (kg), *PL* petiole length (cm), *LLL* length of leaf lobe (cm), *WLL* width of leaf lobe (cm), *RDMC* root dry matter content (%), *NSR* number of storage roots (count), *STC* starch (%), *PH* plant height (cm), *HFB* height at first branching (cm), *NLL* number of leaf lobe (count)^

### Genetic relationship among 102 cassava genotypes using 11 quantitative traits

**Fig. 4:**
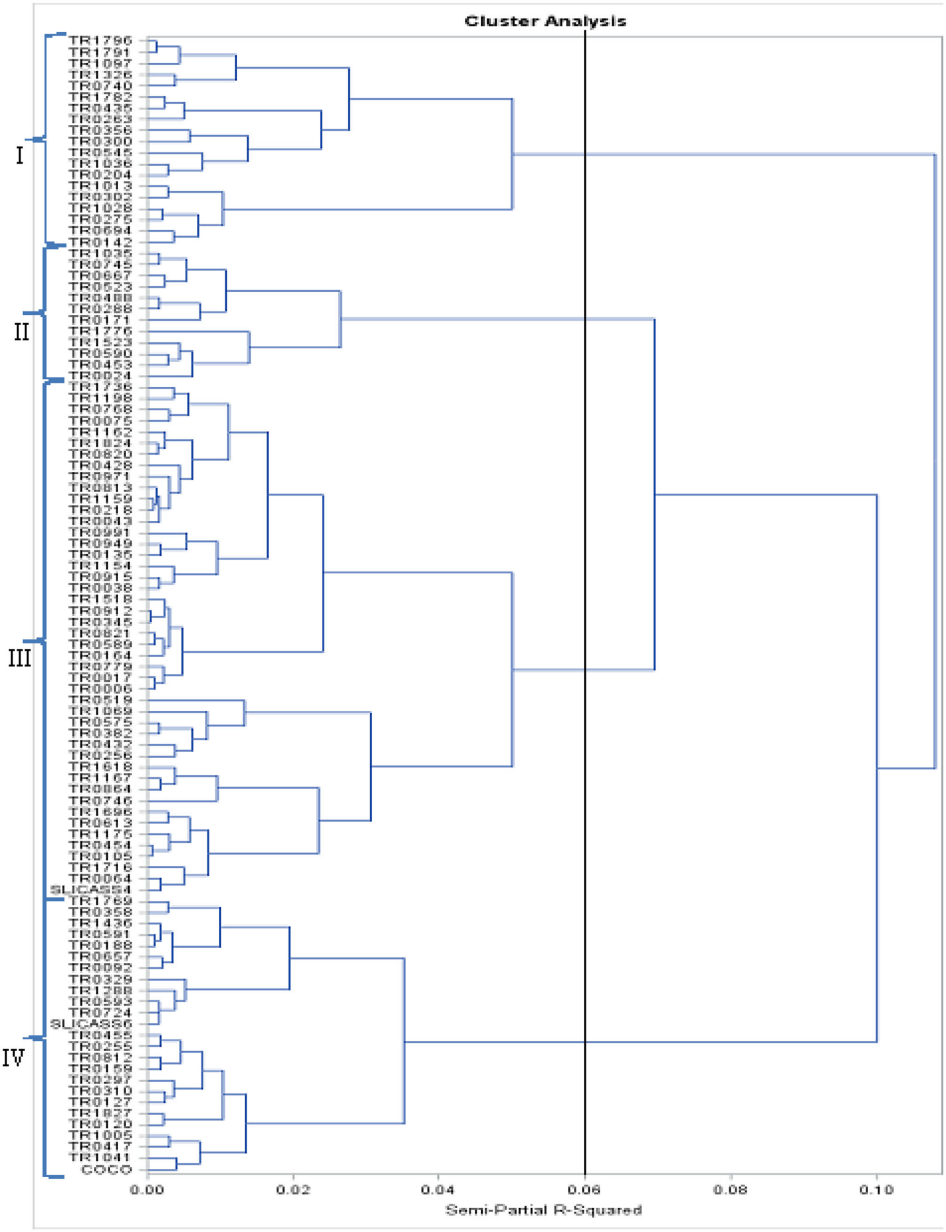
Dendrogram showing relationships among 102 genotypes of cassava classified by Ward method using eleven 11 quantitative agro-morphological traits

Hierarchical classification of quantitative traits grouped genotypes into four classes almost with the same characteristics as a function of the variables (Fig. 4). The genetic similarity for the eleven quantitative traits ranged from zero to one with a mean similarity of 0.10. Cluster I contains 19 genotypes, cluster II 12 genotypes, cluster III 46 genotypes and cluster IV 25 genotypes.

### Clustering analysis using SNPs marker

**Fig. 5:**
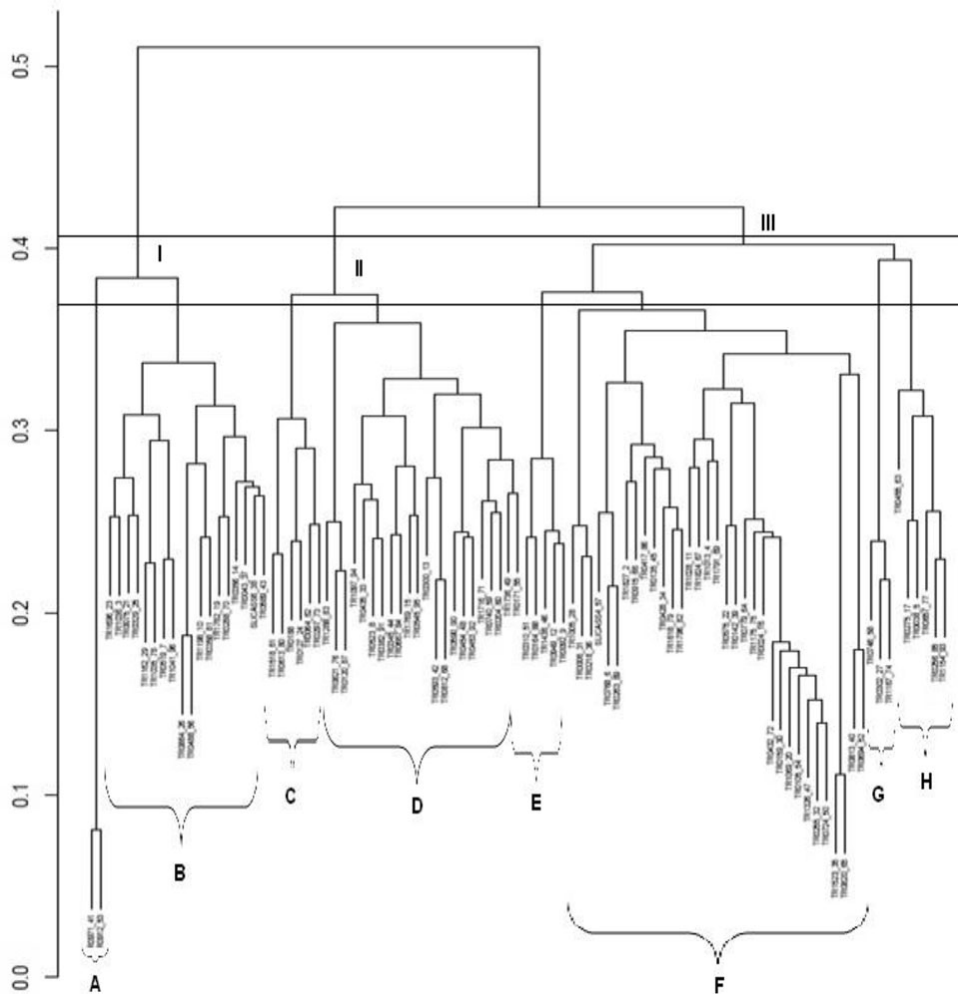
Dendrogram generated by UPGMA method of 96 cassava accessions based on SNP markers

The dendrogram showing clustering analysis of 96 cassava genotypes based on 5600 SNP markers is presented in Fig. 5. At similarity of 0.41, the result revealed three main clusters. At similarity of 0.37, the accessions were further divided into 7 sub-clusters. Cluster I consists of two sub-clusters: sub-clusters A and B. Sub-cluster A had two genotypes (TR0971 and TR0912) and sub cluster B contained 18 accessions. Cluster II consists of two sub-clusters: sub-clusters C and D. Sub-cluster C comprises of 6 accessions; while sub-cluster D contains 22 accessions. Cluster III consists of four sub-clusters: sub-clusters E, F, G and H comprising 5, 33, 3 and 6 accessions, respectively.

## Discussion

The analysis of qualitative morphological traits (root taste, external color of storage root, color of root pulp, ease of peeling, color of leaf vein, cassava mosaic disease, lobe margins, leaf color, color of apical leaves and shape of central leaflet) showed a significant variation among the studied genotypes. Color was apparently the most representative and the most distinctive trait possibly due to the fact that most of the genotypes exhibited white root pulp, and dark brown external storage root. The above-ground leaf attributes of the studied genotypes were light green apical leaves, light green leaf, green leaf vein, smooth lobe margin and pendurate central leaflets. The leaf attributes play key role in cultivar identification and are more important for selection of cassava for the leafy vegetable markets in Sierra Leone where cassava leaves are consumed. These findings concur with Agre et al. (2016) who reported that farmers use the color of the leaves and stems to identify their cassava cultivars.

The principal component analysis is a powerful data reduction technique utilized to reduce large number of correlated variables to a small number that is independent and very useful. The PCA unraveled traits that contributed most to the variation present in the cassava germplasm. The qualitative traits that contributed positively highest to the first PCA include root taste, color of root pulp and ease of peeling. Findings of this study indicate the usefulness of these traits for genotype identification and genetic diversity studies in cassava. These are among key traits often considered relevant for selection of varieties for the genetic improvement of the crop.

The clustering based on similarity index of the qualitative traits in this study grouped the 102 cassava accessions into five clusters. Cluster I contained the accessions characterized by green apical leaves, cluster II grouped accessions having green apical leaves, smooth lobe of leaf margin and resistance to cassava mosaic disease. Cluster III was grouped based on ease of peeling, sweet root taste and conical cylindrical root shape. Cluster IV had light green leaves and green apical leaves. Cluster V contained accessions with dark brown external storage roots, light green leaves and ease of peeling. In a similar study, Raghu et al. (2007) identified six distinct groups using 58 cassava accessions. In this study, the first two principal components explained 31.18% of the total cumulative variance for the qualitative traits. This result is similar with those of Afonso et al. (2014) who found 32.56% of the genetic variance in the first factorial plane. It can also be explained by the fact that the variance distribution is associated with the nature and number of characters used in the analysis and focuses on the first principal components.

The analysis of the 11 quantitative traits revealed significant differences, seven of which had high coefficients of variation. The high coefficients of variation observed for the examined characters indicated the presence of high heterogeneity within the population and therefore can be exploited for breeding. Similar results for cassava were obtained in Benin by Agre et al. (2015) where some averages were identical in cassava diversity study. In this study, starch was positively and highly correlated with dry matter content indicating that starch content and dry matter content are closely related. Similar studies conducted at CIAT and IITA have established that dry matter content and starch content are closely correlated (r = 0.81) (IITA 1974; CIAT 1975).

The first four principal components analysis explained 72.30% of the overall variability in the quantitative analysis. Principal components I, II and III obtained from quantitative variables present yield and yield attribute traits such as harvest index, average yield per plant, number of storage root, root dry matter content and starch content that may be integrated into a cassava breeding program. The quantitative traits with highest positive contribution to distinguishing genotypes in the first PCA included harvest index, average yield per plant, number of storage roots, root dry matter content and starch content. These are among key traits often considered relevant for selection of varieties and for the genetic improvement of the crop. The cluster analysis of the 11 quantitative agro-morphological traits also grouped the accessions into four clusters. Cluster I accessions were characterized by high starch content, root dry matter content and harvest index. Cluster II accessions were characterized by high root dry matter content and fresh storage root yield. Cluster III accessions exhibited high starch content; whilst cluster IV accessions contained high root dry matter content. The results generally indicate the relevance of the above yield and yield attribute traits in characterizing the genotypes. It also depicts the usefulness of the agro-morphological descriptor by Fukuda et al. (2010) in identifying variability and reducing dimensionality in the traits set. In this study, the 11 qualitative and 11 quantitative trait sets sufficiently discriminated the 102 genotypes into distinct cluster groups. All accessions differ from each other in one or more traits with no detection of duplicates, which suggest their usefulness in genotypic differentiation and identification.

Findings of the molecular study revealed that 96% of the 5600 SNP markers were polymorphic. The highest polymorphic information content (PIC) value observed was 0.17. The variation observed reflect the genetic constitution of the accessions. In a previous study on cassava genetic diversity using SNPs, Kawuki et al. (2009) reported PIC values of 0.228 in 74 cassava accessions. Moreover, in maize, Yang et al. (2011) reported a higher PIC value of 0.34 using 884 SNP markers. The variance in PIC values among these studies could be attributed to the number of genotypes and type of SNP markers used. Both the morphological and SNP markers established the uniqueness and variability within the cassava germplasm utilized in this study. The unique diversity in the cassava germplasm suggests that the germplasm might possess genes, in high frequencies, for adaptation in the studied area, whereas the high genetic diversity is indicative of a high amount of additive genetic variance, needed for genetic progress in plant breeding. The high genetic variability also represents a heterotic pool that provides an opportunity for the systematic exploitation of hybrid vigor in cassava. Although high diversity has been noted for African cassava germplasm (Lyimo et al. 2012), however, such diversity is still lower than those observed in Southern America cassavas (Hurtado et al. 2008; da Silva et al. 2015). Unlike the later where farmers use seedling and vegetative propagation techniques (Siqueira et al. 2009; Mezette et al. 2013), farmers in Sierra Leone only propagate the crop using stem cuttings. This study established the true-to-type genetic identity and useful variability within cassava germplasm of Sierra Leone needed for the genetic improvement and conservation of the crop.

## Conclusion

This study successfully determined the extent of genetic diversity within cassava breeding population of Sierra Leone using morphological and SNP markers. It also provides a data base for cassava breeders to make informed decision for parental selection in a cassava breeding program based upon genetic diversity. The useful genetic variability for storage root number, starch content, root dry matter content and storage root yield that were identified could be exploited for the genetic improvement of the crop and its conservation. The color attribute of various qualitative traits studied contributed most to the differentiation of genotypes. The agro-morphological and SNP marker techniques were complementary in distinguishing the cassava genotypes. Both approaches should therefore be used for genetic diversity studies of cassava.

## References

[CR1] Afonso SDJ, Ledo CAdaS, Moreira RFC, Silva SdeOe, Leal VDdeJ, Conceição ALdaS (2014) Selection of descriptors in a morphological characteristic considered in cassava accessions by means of multivariate techniques. IOSR-JAVS 7: 13–20

[CR2] Agre AP, Dansi A, Rabbi IY, Battachargee R, Dansi M, Melaku G, Augusto B, Sanni A, Akouegninou A, Akpagana K (2015). Agro morphological characterization of elite cassava (*Manihot esculenta* Crantz) cultivars collected in Benin. Int J Curr Res Biosci Plant Biol.

[CR3] Agre AP, Badara G, Adjatin A, Dansi A, Bathacharjee R, Rabbi IY, Dansi M, Gedil M (2016). Folk taxonomy and traditional management of Cassava (*Manihot esculenta* Crantz) Diversity in Southern and Central Benin. Int J Innov Sci Res.

[CR4] Andrade EKV, Andrade Júnior VC, Laia ML, Fernandes JSC, Oliveira AJM, Azevedo AM (2017). Genetic dissimilarity among sweet potato genotypes using morphological and molecular descriptors. Acta Sci Agron.

[CR5] Asare PA, Galyuon IKA, Sarfo JK, Tetteh JP (2011). Morphological and molecular based diversity studies of some Cassava (*Manihot esculenta* Crantz) Germplasm in Ghana. Afr J Biotech.

[CR6] Beeching JR, Marmey P, Gavalda MC, Noirot M, Haysom HR, Hughes MA, Charrier A (1993). An assessment of genetic diversity within a collection of cassava (*Manihot esculenta* Crantz) germplasm using molecular markers. Ann Bot.

[CR7] Benesi M (2005) Characterization of Malawian Cassava Germplasm for diversity, Starch extraction and its Native and modified properties. PhD thesis, Free State University, South Africa

[CR8] Bradbury PJ, Zhang Z, Kroon DE, Casstevens TM, Ramdoss Y, Buckler ES (2007). TASSEL: software for association mapping of complex traits in diverse samples. Bioinformatics.

[CR9] CIAT (1975) Centro International de Agricultural Tropical (CIAT) Cali, Colombia. Annual Reports, 1974

[CR10] Collard BCY, Jahufer MZZ, Brouwer JB, Pary ECK (2005). An introduction to markers, quantitative trait loci (QTL) mapping and marker-assisted selection for crop improvement: the basic concepts. Euphytica.

[CR11] da Silva LI, Filho PSV, da Costa TR, Domingos ML, Goncalves-Vidigal MC (2015). Molecular characterization of traditional Assessions from the periurban region, Toledo, Western Parana, Southern Brazil. J Glob Biosci.

[CR12] Dellaporta SL, Wood J, Hicks JB (1983). A plant DNA mini preparation Version II. Plant Mol Biol Rep.

[CR13] Dixon AGO, Ngeve JM, Nukenine EN (2002). Genotype × environment effects on severity of cassava bacterial blight disease caused by *Xanthomonas axonopolis* pv. *manihotis*. Eur J Plant Pathol.

[CR14] Elias M, McKey D, Panaud O, Anstett MC, Robert T (2001). Traditional management of cassava morphological and genetic diversity by the Makushi Amerindians (Guyana, South America): perspectives for on-farm conservation of crop. Euphytica.

[CR15] Elshire RJ, Glaubitz JC, Sun Q, Poland JA, Kawamoto K, Buckler ES, Mitchell ES (2011). A robust, simple genotyping-by-sequencing (GBS) approach for high diversity species. PLoS ONE.

[CR16] Ferri L, Perrin E, Campana S, Tabacchioni S, Taccetti G, Cocchi P, Ravenni N, Dalmastri C, Chiarini L, Bevivino A, Manno G, Mentasti M, Fani R (2010). Application of multiplex single nucleotide primer extension (mSNuPE) to the identification of bacteria: the *Burkholderia cepacia* complex case. J Microbiol Methods.

[CR17] Fregene M, Bernal A, Duque M, Dixon AGO, Tohme J (2000). AFLP analysis of African cassava (*Manihot esculenta* Crantz) germplasm resistant to the cassava mosaic disease (CMD). Theor Appl Genet.

[CR18] Fukuda WMG, Guevara CL (1998) Morphological and agronomic descriptors for the characterization of manioc (*Manihot esculenta* Crantz). Cruz das Almas: Embrapa Cassava and Fruticulture, Documents 78

[CR19] Fukuda WMG, Guevara CL, Kawuk R, Ferguson ME (2010) Selected morphological and agronomic descriptors for the characterization of cassava. International Institute of Tropical Agriculture (IITA): Ibadan (Nigeria)

[CR20] Grattapaglia D, Kirst M (2008). Eucalyptus applied genomics: from gene sequences to breeding tools. New Phytol.

[CR21] Hurtado P, Olsen KM, Buitrago C, Ospina C, Marin J, Durque M, de Vincente C, Wongtiem P, Wenzel P, Killian A, Adeleke M, Fregene M (2008). Comparison of simple sequence repeat. Plant Genet Resour.

[CR22] IITA (1974). Cassava annual report.

[CR23] IITA (1990). Annual report.

[CR24] Kawuki R, Ferguson M, Labuschagne M, Herselman L, Kim DJ (2009). Identification, characterisation and application of single nucleotide polymorphisms for diversity assessment in cassava (*Manihot esculenta* Crantz). Mol Breed.

[CR25] Liu K, Muse SV (2005). Power marker: integrated analysis environment for genetic marker data. Bioinformatics.

[CR26] Mckey D, Emperaire L, Elias M, Pinton F, Robert T, Desmoulière S, Rival L (2001). Local management and regional dynamics of varietal diversity of cassava in the Amazon. Genet Sel Evol.

[CR27] Mezette TF, Blumer CG, Veasey EA (2013). Morphological and molecular diversity among cassava genotypes. Pesq Agropec Bras.

[CR28] Nassar NMA (2005) Cassava: some ecological and physiological aspects related to plant breeding. An article published online with Gene Conserve

[CR29] Okogbenin E, Egesi CN, Olasanmi B, Ogundapo O, Kahya S, Hurtado P, Marin J, Akinbo O, Mba C, Gomez H, de Vicente C, Baiyeri S, Uguru M, Ewa F, Fregene M (2012). Molecular marker analysis and validation of resistance to cassava mosaic disease in elite cassava genotypes in Nigeria. Crop Sci.

[CR30] Park Y-J, Lee JK, Kim NS (2009). Simple sequence repeat polymorphisms (SSRPs) for evaluation of molecular diversity and germplasm classification of minor crops. Molecules.

[CR31] Pinton F, Emperaire L (2001). Cassava in the Brazilian Amazon: varietal diversity and market. Genet Sel Evol.

[CR32] Rabbi I, Hamblin M, Gedil M, Kulakow P, Ferguson M, Ikpan AS, Ly D, Jannink J-L (2014). Genetic mapping using genotyping-by-sequencing in the clonally propagated cassava. Crop Sci.

[CR33] Raghu D, Senthil N, Saraswathi T, Raveendran M, Gnanam R, Venkatachalam R, Shanmugasundaram P, Mohan C (2007). Morphological and simple sequence repeats (SSR) based finger printing of South Indian cassava germplasm. Int J Integrat Biol.

[CR34] Richman MB (1988). A cautionary note concerning a commonly applied eigenanalysis procedure. Tellus.

[CR35] Sánchez T, Salcedo E, Ceballos H, Dufour D, Mafla G, Morante N, Calle F, Perez JC, Debouck D, Jaramillo G, Moreno IX (2009). Screening of starch quality traits in Cassava (*Manihot esculenta* Crantz). Starch/Stärke.

[CR36] Scott GJ, Rosegrant MW, Ringler C (2000) Roots and tubers for the 21st century: trends, projections, and policy options. No 31, 2020 vision discussion papers, International Food Policy Research Institute (IFPRI). https://econpapers.repec.org/paper/fpr2020dp/31.htm

[CR37] Siqueira MVBM, Queiroz Silva JR, Brascan EA, Borges A, Pereira KJC, Pinto JG, Veasey EA (2009). Genetic characterization of cassava (*Manihot esculenta*) landraces in Brazil assessed with simple sequence repeats. Genet Mol Biol.

[CR38] Smykal P, Horacek J, Dostalova R, Hybl M (2008). Variety discrimination in pea (*Pisum sativum* L.) by molecular, biochemical and morphological markers. J Appl Genet.

[CR39] Souza DCL (2015). Molecular techniques for characterization and conservation of medicinal and aromatic plants: a review. Rev Bras Plantas Med.

[CR40] Temegne NC, Ajebesone FN, Kuate AF (2015). Influence of soil chemical composition on nutrient content and yield of cassava (*Manihot esculenta* Crantz, Euphorbiaceae) in two agro-ecological zones of Cameroon. Int J Biol Chem Sci.

[CR41] Yang J, Dong A, Peng Z (2011). Expression profiling of cassava storage roots reveals an active process of glycolysis/glyconeogenesis. J Integrat Plant Biol.

